# 

**DOI:** 10.1192/bjb.2022.66

**Published:** 2023-08

**Authors:** Will Smith

**Affiliations:** is a Foundation Doctor at Gloucestershire Hospitals NHS Foundation Trust, UK. Email: w.smith1@doctors.org.uk


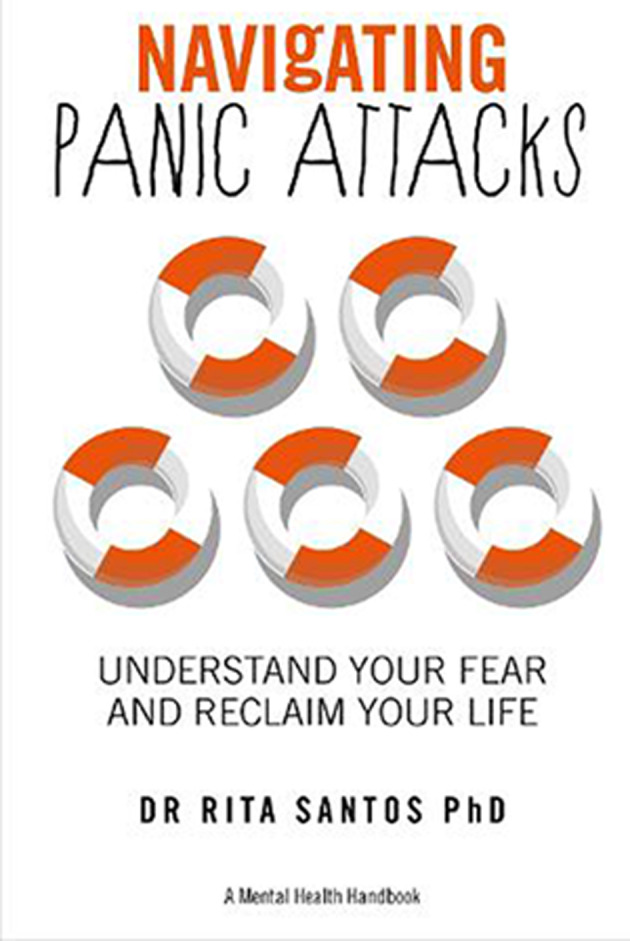


In presenting her guidance in *Navigating Panic Attacks*, Dr Rita Santos pitches an army of feelings against an army of knowledge and attempts to fortify the latter. By strengthening the ‘rational’ army of knowledge with information and understanding, a person may overcome panic attacks (ruled by emotions and irrationality) and reclaim heir life, Santos argues.

In this way, the book sets about imparting knowledge about both the physiology and personal relevance of panic attacks, in a manner that is notably pitched for universal accessibility. In Santos's own words, ‘I like to simplify’; and she is excellent at it. She transparently explains with ease what exactly panic attacks are, including the signs, phenomenology and physiology, going on to explore how sufferers respond maladaptively and offering guidance on mediating this response to adopt better coping strategies. The book even features pull-quotes on almost every page, presumably to further aid accessibility or pace of consumption for those with busy lives. In opting for simplicity in these explanations, however, there is the sense that those who prefer further detail or have a background in mental health might want to look elsewhere. With Santos's extensive background in research, I personally would have enjoyed learning more about the complexities of panic attacks, and sufferers of panic attacks, many of whom will be experts by experience, may feel the same.

The book does draw directly on the experiences of people who suffer from panic attacks, though, offering numerous examples and even direct quotes from patients, which no doubt serve as a tool for empowerment and reducing stigma. It also features exercises throughout, which encourage journalling, personal reflection and reappraisal. The book reaches its climax when Santos offers her golden advice on responding to panic attacks – which is to do nothing at all, allowing them simply to run their course. Although counter-intuitive, Santos offers mindful and progressive strategies for achieving this aim and provides the glowing feedback of people who have effectively adopted the methods she describes.

Throughout the book, Santos adopts the tone of an informal podcaster or a learned friend, which serves as an antidote for clinical paternalism much maligned by patients. With this in mind, together with her credentials, I have no doubt this book would be an excellent tool to offer patients who want to understand more about their condition or as an adjunct to therapy. However, after reading the book, I was left with an underlying sense of confusion about the exact intended purpose of such books (this is one of a set of four ‘mental health handbooks’ on navigating common problems). I hope that readers of Santos's book might reap just a fraction of benefit that patients would have, had they had access to the service in the flesh.

